# Collagenase *Clostridium histolyticum* for the Treatment of Cellulite in the Buttocks and Thigh: Early Insights From Clinical Practice

**DOI:** 10.1093/asjof/ojac057

**Published:** 2022-06-29

**Authors:** Miles Graivier, David Hill, Bruce Katz, Kristin A Boehm, Juliya Fisher, China Battista

## Abstract

**Background:**

Collagenase *Clostridium histolyticum* (CCH-aaes; QWO [Endo Aesthetics, Malvern, PA]) is an injectable, enzyme-based treatment indicated for the treatment of moderate to severe cellulite on the buttocks of adult women. The minimally invasive nature of the treatment makes it an attractive option for targeted disruption of the fibrous septae which give rise to the dimples characteristic of cellulite in buttocks and thighs.

**Objectives:**

The article provides an overview of cellulite treatment with CCH-aaes, including patient identification and education, treatment planning, CCH-aaes dilution, injection technique, safety, and early experience with mitigation of adverse events, including bruising.

**Methods:**

As part of a continuing medical education (CME; xMedica, Alpharetta, GA) event on developments in cellulite treatment, a panel of experts developed a course and roundtable, which included lectures on cellulite physiology, new developments in the field of cellulite treatment, demonstrations of injection technique for CCH-aaes, and a review of considerations for the use of CCH-aaes in real-world clinical practice.

**Results:**

The practical guidance presented here is based upon real-world experience with CCH-aaes. The discussion includes strategies based on early experience for how to obtain the best results as well as suggestions on how to mitigate bruising.

**Conclusions:**

CCH-aaes has been a welcome addition to the armamentarium for the treatment of cellulite. With knowledge of proper patient evaluation and injection technique, thorough patient education, diligent photography, and developing research on bruising mitigation, CCH-aaes shows great promise as an effective and safe modality for the management of cellulite.

**Level of Evidence: 5:**

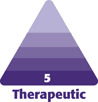

Collagenase *Clostridium histolyticum* (CCH-aaes; QWO [Endo Aesthetics, Malvern PA]) is a targeted, enzyme-based treatment approved by the FDA for the treatment of moderate to severe cellulite on the buttocks of adult women.^1^ Administered as a subcutaneous injection, CCH-aaes is composed of a 1:1 ratio of 2 collagenases: collagenase I (AUX-I, Clostridial class I collagenase) and collagenase II (AUX-II; Clostridial class II collagenase), which cleave type I and III collagen in a specific fashion.^2,3^ When injected into a cellulite dimple, CCH-aaes disrupts the fibrous septae tethered to the underside of the dermis, thereby alleviating the local tension thought to cause the skin topography characteristic of cellulite.^4-6^

Cellulite itself is a multifactorial condition, with skin laxity and/or thickness as well as patient body weight potentially exacerbating its appearance.^4,7^ While a number of hypotheses exist as to the true underlying physiologic cause of the condition, the actual appearance of cellulite is thought to be a function of the type (thickness and organization) of fibrous septae, the capacity of the dermis to support the underlying tissues, and the architecture of both the deep and superficial fat layers.^8^ It is the loss of mechanical balance between these factors that dictates whether cellulite is apparent on the skin’s surface. Importantly, while laxity and adiposity contribute to the global appearance of the buttock and thigh area, they must be differentiated from true cellulite dimpling in order to assure appropriate treatment and optimal outcome. The central role of fibrous septae becomes apparent when assessing the relative efficacy and durability of alternate treatment modalities for the management of this condition. Treatments aimed exclusively at laxity (eg, energy-based devices or hyperdilute biostimulatory fillers) or adiposity (eg, liposuction or cryolipolysis) often have an inconsistent or temporary impact and may even exacerbate the underlying condition. Surgical subcision is a more effective treatment, as it directly addresses the fibrous septate, but is by definition an invasive therapy associated with discomfort and side effects that limit its widespread adoption.^4^ CCH-aaes represents both a targeted and minimally invasive option that allows for disruption of the fibrous septae and resolution of cellulite dimples in the buttocks and thighs (a cuttently off-label area). The familiarity of injection, minimal patient discomfort during the procedure, and short timeline to efficacy render CCH-aaes a welcome addition to the current collection of available treatments for managing this condition.

## METHODS 

As part of a continuing medical education (CME) event on developments in cellulite treatment (xMedica, Alpharetta, GA), a panel of experts in the treatment of cellulite was convened. The panel included 3 plastic surgeons, 2 board-certified dermatologists (2 of these faculty were investigators for CCH-aaes clinical studies), and 1 nurse practitioner injector. The faculty developed 3 hours of virtual webinars, including lectures on cellulite and new developments in the field, demonstrations of injection technique for CCH-aaes, and a review of proper patient selection. The meeting culminated in a 1-hour roundtable discussing the use of CCH-aaes in real-world clinical practice. The series was held at professional conferences and published online from September 2021 through March 2022. Through discussion, topics for this manuscript were identified by faculty, including patient identification and education, treatment planning and dilution, injection technique, safety, and mitigation of adverse events (AEs), including bruising. All patients discussed were treated in accordance with the principles outlined in the Declaration of Helsinki, and each patient consented to treatment and photography.

## RESULTS

The CME series was held at aesthetics-focused medical conferences and published online from September 2021 through March 2022. Through discussion, topics for this manuscript were identified by the faculty, including patient identification and education, treatment planning and dilution, injection technique, safety, and mitigation of AEs, including bruising. Together, the panelists have treated well over 350 patients with CCH-aaes both within the context of clinical trials and in clinical practice. While on-label use of CCH-aaes is currently restricted to cellulite on the buttocks, the discussion also included off-label treatment of the thighs. All patients discussed were treated in accordance with the principles outlined in the Declaration of Helsinki, and each patient consented to treatment and photography.

## DISCUSSION

### Patient Selection and Education

Because injectables are widely recognized and accepted as both effective and minimally invasive, patients generally are comfortable with the idea of injections for cellulite, and interest in treatment in clinical practice has been high. While diligent patient selection is key to the success of all aesthetic treatments, an accurate diagnosis of cellulite and the relative contribution of laxity and skin quality issues to its appearance is critical. First, the treatment of cellulite will not mitigate unevenness in topography caused by laxity. Anecdotally, treatment of cellulite without proper management of contributing laxity may actually worsen overall appearance, as release of multiple points of tension removes local effects that may have held the skin somewhat taut. Furthermore, educating the patient on the difference between laxity (due to skin quality or volume loss) and cellulite, and the relative contribution of each to their global appearance, is essential for setting realistic patient expectations and and initiating any needed conversation around combination treatment with other modalities and why they may be needed for an optimal result. It is also crucial to obtain a detailed medical history, as contour irregularities may be secondary to previous procedures and interventions performed by other practitioners (eg, liposuction).

While there is no age limit for the treatment of cellulite, the patients who respond best to CCH-aaes are generally younger with excellent skin elasticity and without compounding skin laxity. These patients often experience an excellent response regardless of the baseline severity of their condition. Importantly, however, even in patients with good skin quality, a very high BMI may make results difficult to appreciate given the independent contribution of localized adiposity to skin surface irregularity.

Response to treatment in patients with excessive sun damage, which is common on the backs of the thighs, has not been as good, even in those with minimal additional laxity. The basis for this lack of response is not known, but one hypothesis is that the smoothing effect of CCH-aaes treatment is in part due to the induction of the healing response initiated by the cleaving of resident collagen and that this element of treatment response is not as robust in these patients.

#### Treatment Planning

Most patients in clinical practice present with cellulite in both the buttocks and thighs. When treating these areas, several factors demand careful pretreatment planning. First, the patient must be evaluated in the standing position, in both a relaxed and clenched state, so that dimples can be identified and marked (Video 1). Once the patient is placed in the prone position for injection, dimples are difficult to identify. Second, the CCH-aaes dilution used to treat the buttock (0.23 mg/mL) is different from that used for the thigh (0.046 mg/mL, a 1:5 dilution using normal saline, not bacteriostatic saline). If treating both the buttocks and the thigh, the dimples must be identified ahead of time so that the proper dilution can be made. Of note, in one author’s practice, it is rare to split a vial between the buttock and the thighs. Rather, the buttock is treated with a dedicated vial and the thigh with a separate dedicated vial.

The number of buttock dimples that can be treated is dependent upon the size of the vial. The calculations below assume some loss of volume during reconstitution and/or draw up of product: 


$$\begin{array}{ll} & Up\;to\;12\;dimples\;can\;be\;treated\;with\;a\;4\text{-}mL\;vial:\\ & \;0.3\;mL\;per\;dimple\;*\;12\;dimples=3.6mL \end{array}$$

$$\begin{array}{ll} & Up\;to\;24\;dimples\;can\;be\;treated\;with\;an\;8\text{-}mL\;vial:\\ & \;0.3mL\;per\;dimple\;*\;24\;dimples=7.2mL \end{array}$$

Once the number of desired buttock dimples has been identified, calculations are needed to determine the volume of diluent needed so that remainder of the vial can be properly prepared to treat the thighs. To treat the thighs, reconstituted CCH-aaes (0.23 mg/mL) should be diluted 1:5 (1 part CCH-aaes, 4 parts diluent) to achieve the 0.046 mg/mL concentration needed for treatment.^9^ While this calculation can be done ahead of time, a straightforward way to measure the remaining volume of QWO mid-treatment is to draw up the remaining product into a syringe to measure the volume, then multiply the remaining volume by 4 to obtain the volume of diluent needed.

The following calculation can be used to determine the diluent needed to treat the thighs:


$$\begin{array}{ll} & 4\;mL\;-\;\left(number\;of\;buttock\;dimples\;x\;\;0.3\right)=\\ & \;volume\;\left(mL\right)\;of\;remaining\;CCH\;\text{-}\;aaes \end{array}$$

or


$$\begin{array}{ll} & 8\;mL-\left(number\;of\;buttock\;dimples\;x\;0.3\right)=\\ & \;volume\;\left(mL\right)\;of\;remaining\;CCH\;\text{-}\;aaes \end{array}$$

With the volume of remaining CCH-aaes in hand, the volume of diluent needed to treat the thigh can be calculated:


$$\begin{array}{ll} & volume\;\left(mL\right)\;of\;remaining\;CCH\text{-}aaes*4=volume\;\left(mL\right)\;\\ & \;of\;diluent\;to\;be\;added\;to\;remaining\;CCH\;\text{-}\;aaes \end{array}$$

As the volume of diluent needed will likely be more than what can be accommodated by the vial, the CCH-aaes to be diluted may be added to a sterile container containing the desired volume of normal saline. The easiest way to accomplish this is to use a bottle of sterile saline (20 or 50 mL) with an appropriate volume removed. For example, if 16 mL of diluent is needed, then a 20-mL bottle of sterile saline can be used, with 4 mL removed before the addition of the CCH-aaes.

The number of thigh dimples that can be treated is equal to the total volume of diluted CCH-aaes divided by the 1.5 mL needed to treat each dimple:


$$\begin{array}{ll} & (volume\;of\;remaining\;CCH-aaes\;x\;\;5)/\;1.5\;mL\;=\;\\ & \;thigh\;dimples\;that\;can\;be\;treated \end{array}$$

When reconstituting CCH-aaes, it is important to use the solution that comes with the product, as it contains ions that are important for the function of the enzyme.^10^ For dilution, it is important to use normal saline rather than bacteriostatic saline. Laboratory data evaluating the activity, purity, and solubility of CCH-aaes have shown that bacteriostatic saline can cause up to a 40% loss in activity and aggregation of 5.3% of the protein, while normal saline does not have this effect.^10^

Finally, pretreatment planning should involve standardized photography of the patient. Photographs should be taken from 0°, 22°, and 90° with the patient in the relaxed and clenched position. It is also important to take images of the patient before marking dimples to be treated and after the markings are made, so that the individual dimples injected are highlighted. While the highest standards of clinical photography for cellulite can involve advanced equipment and a high degree of technical expertise, for the practical purposes of documenting treatment effect for the patient, the most important aspect of photography is consistency.^11^ Consistent background and lighting, distance from the camera (especially important for cameras with fixed focal length like tablets), and clothing (a black G-string can be provided) facilitate comparison of the before and after images. In some instances, using the black and white mode for the camera can highlight shadowing caused by dimples, making them easier to see. Attention to lighting is particularly important as small changes in light direction and intensity can affect the appearance of shadows. For evaluating the impact of treatment, photographs can be taken before each treatment session with and without markings and in the relaxed and clenched positions. However, if the goal is to evaluate bruising, the trajectory of bruising demands more frequent photography with the patient returning for follow-up photography between treatments.

Clinical photography is especially important given the potential need for countering perceptual drift and recall bias over time.^12^ In the CCH-aaes phase 3 studies RELEASE-1 and RELEASE-2, a 37.1% and 47.6% composite response rate (≥1-level severity improvement from baseline in both Clinician Reported Photonumeric Cellulite Severity Scale [CR-PCSS] and Patient-Reported Photonumeric Cellulite Severity Scale [PR-PCSS] ratings at day 71) was reported; however, subjects did not evaluate their cellulite in comparison to baseline photographs but instead were asked to base their score on their cellulite at the time of evaluation. On day 71 in RELEASE-1, 54.3% were “satisfied” or “very satisfied” with their treatment, and, in RELEASE-2, 46.8% of patients were at least satisfied.^13^ In clinical practice, the satisfaction of patients with treatment is generally higher. This is likely due, in part, to the personalized patient selection that occurs in real-world practice (which is informed by early experience with clinical trials), but also because of the ability of patients to see their baseline photographs and compare them to photographs from posttreatment time points. While not fully quantified with real-world data, this disparity highlights the importance of both photography and patient selection.

Anecdotally, one treatment effect that is not reported in studies but is apparent in clinical practice is a local smoothing effect in patients without significant laxity. Though the most efficient action of CCH-aaes is local, there can be a wider field of effect (3-5 cm from the site of injection in some patients), in particular in the thighs where dimples are oblong and less discrete. Whether this is related to small differences in the depth of injection and/or diffusion of the CCH-aaes enzyme itself or a secondary wound-healing effect is unclear, and the mechanism of this effect remains unknown. Further research in this area will likely define not only the field of effect but also the basis for the large surface area of bruising when it does occur.

### Injection Technique

**Figure 1.**
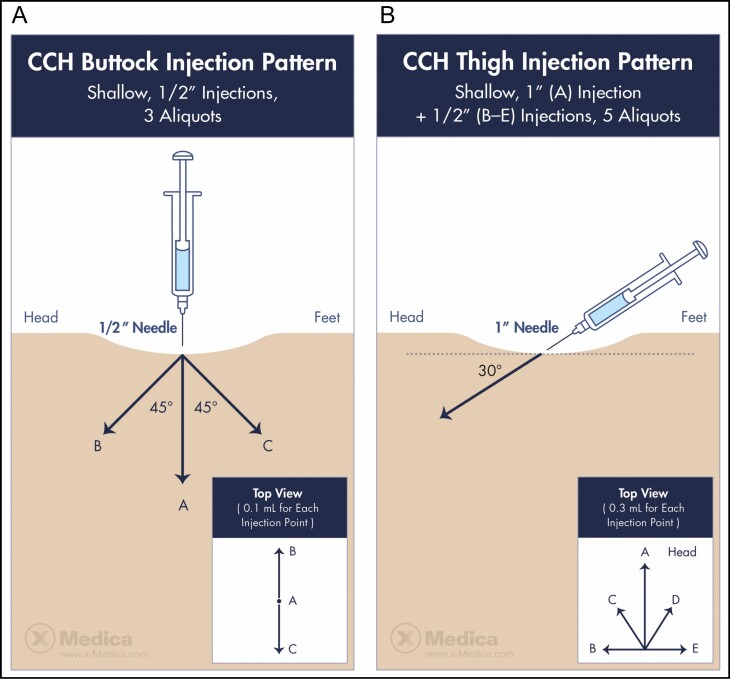
Injection patterns for collagenase *Clostridium histolyticum* in the (A) buttock and (B) thigh. Images published with permission from James Silvera and xMedica, LLC.

Before the initiation of phase 3 clinical trials, 5 injection techniques were studied in both the buttock and thigh to assess the efficacy and safety.^9^ Of those, a 3-aliquot injection (three 0.1-mL injections of 0.23 mg/mL CCH-aaes) was identified as optimal for the buttock and later used in phase 3 pivotal studies (Figure 1A).^9,13^ Importantly, a different injection technique was identified as optimal for injection in the thigh (Figure 1B), which includes 5 injections of 0.3 mL of 0.046 mg/mL of CCH-aaes (a 1:5 dilution of the concentration used in the buttock) in a fan-like distribution.^9^ Each of the authors has largely adhered to these 2 techniques but noted that additional studies may lead to further refinement. Step-by-step injection of the buttock is shown in Video 2, and injection of the thighs is shown in Video 3.

### Treatment Interval and Timing

**Figure 2.**
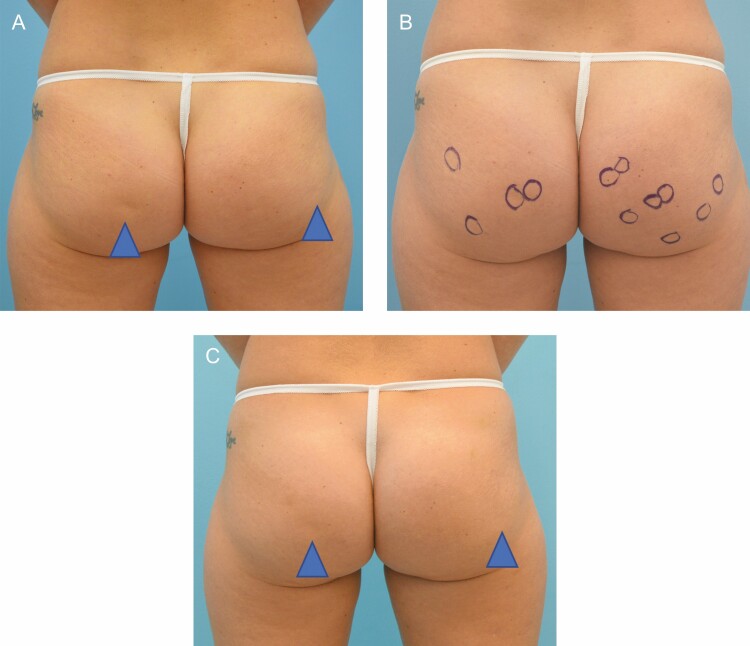
A 32-year-old female at baseline (A, B) and 13 weeks (C) following her first treatment with collagenase *Clostridium histolyticum* to the buttocks.

**Figure 3.**
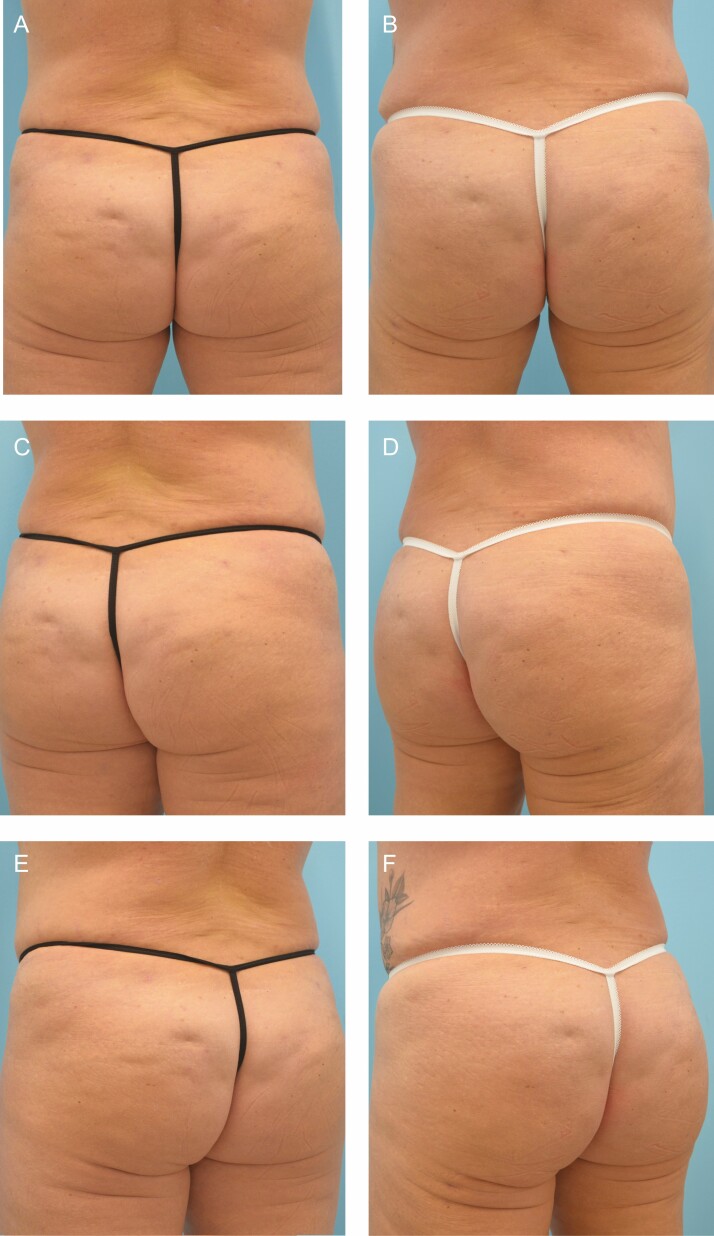

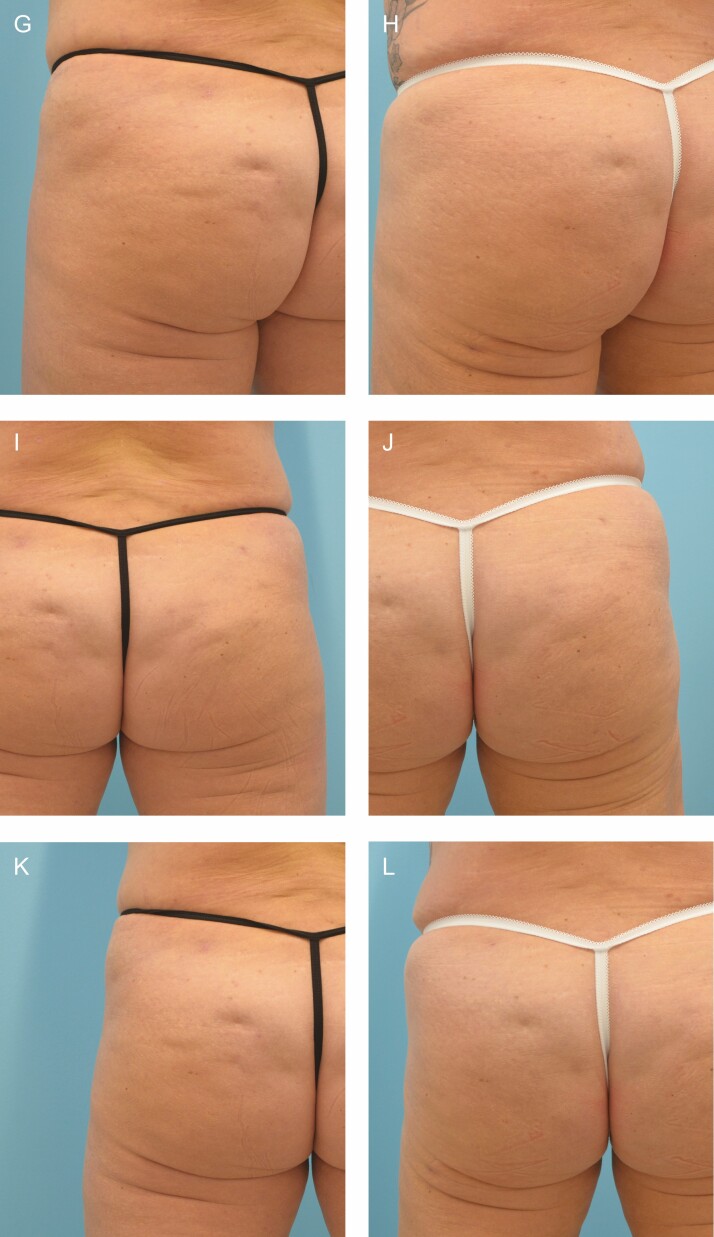
A 46-year-old female at baseline (A, C, E) and 31 weeks after (B, D, F) 4 treatments with collagenase *Clostridium histolyticum* in the buttocks and posterior thighs. Close up before (G, I, K) and after (H, J, L) images are also shown.

**Figure 4.**
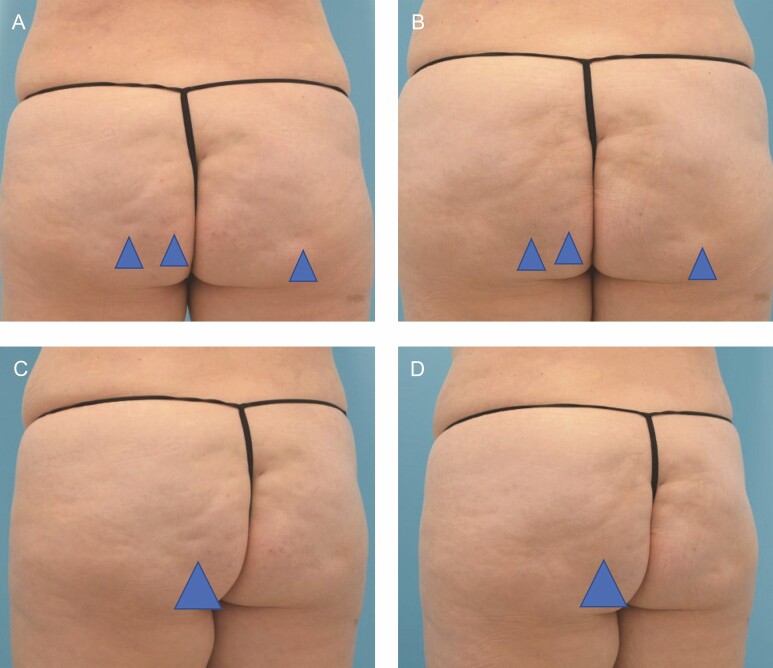

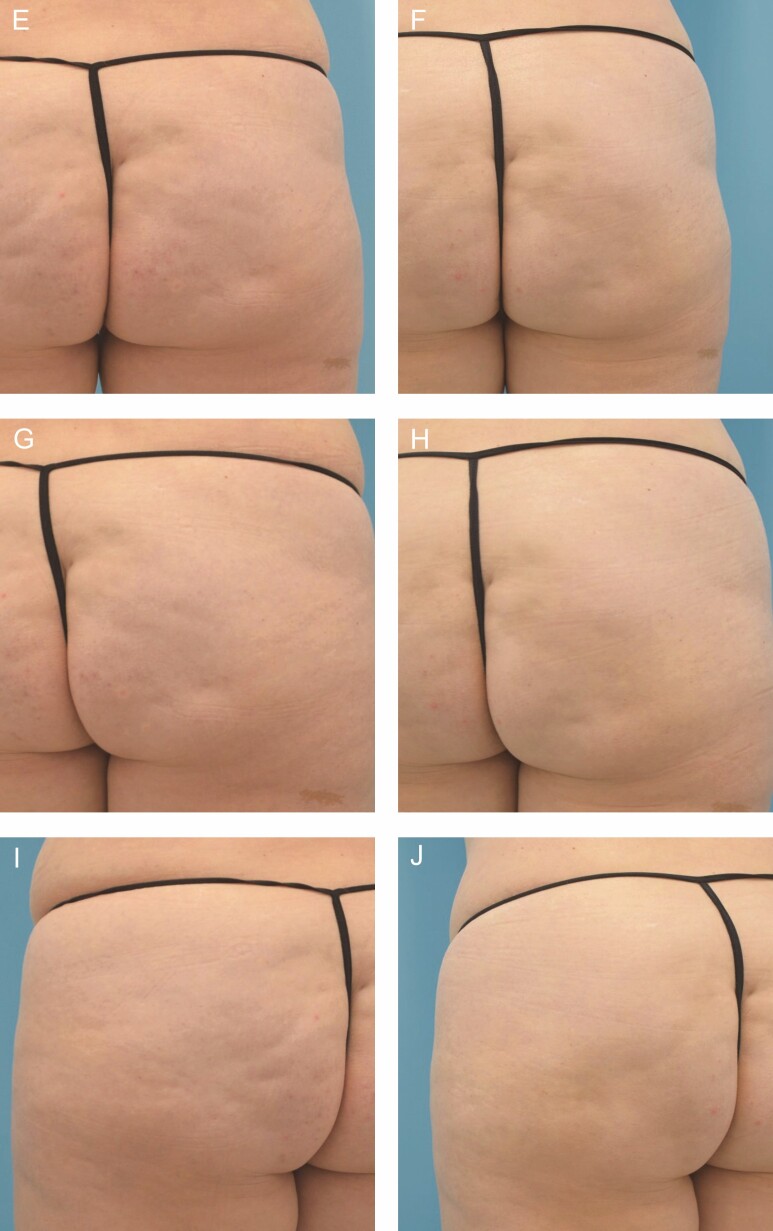
A 51-year-old female at baseline (A, C) and 36 weeks after (B, D) 3 treatments with collagenase *Clostridium histolyticum* in the buttocks. Close up before (E, G, I) and after (F, H, J) images are shown.

**Figure 5.**
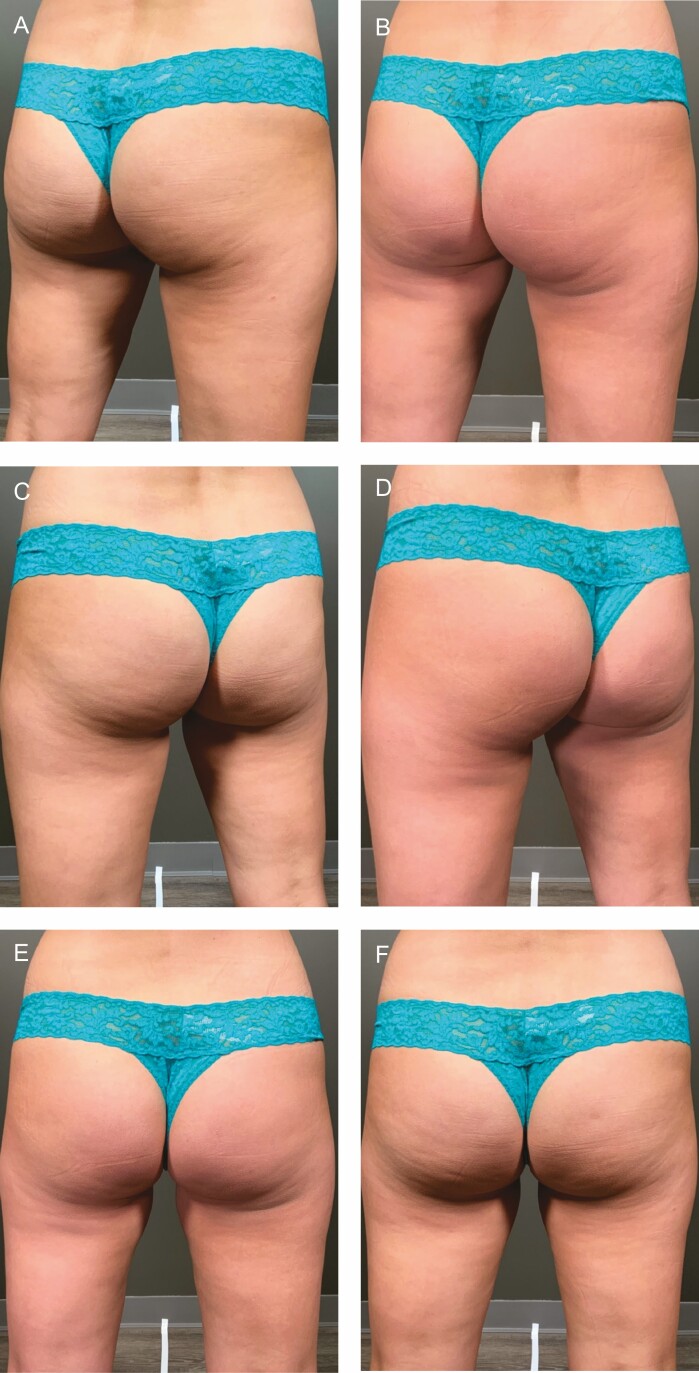
A 39-year-old female at baseline (A, C, E) and 4 months after initiating treatment with collagenase *Clostridium histolyticum* (B, D, F). After photographs were taken 47 days following her third treatment session.

In clinical studies of CCH-aaes, treatment is administered across 3 injection sessions 21 days apart.^13^ Thus far in clinical practice, this treatment schedule has been acceptable for patients and has permitted the dissipation of bruising and swelling such that the patient has fully recovered by the subsequent treatment session. Consistent with observations in clinical studies in both the buttock and thigh, the duration and severity of bruising are reduced with each subsequent session.^13,14^ While some increase in interval to accommodate patient scheduling or to permit complete reduction of swelling is reasonable, anecdotal experience is that, if multiple weeks are allowed to pass, the severity and duration of bruising in the subsequent session are most like that observed with the initial treatment. Though response is generally apparent after 1 session (Figure 2), and some patients are satisfied after 2 sessions, most require all 3 treatments. Representative treatment outcomes are shown in Figures 3-5.

To date, none of the panelists have treated patients with more than 4 sessions, and thus far, retreatment has not been needed for maintenance or the reemergence of previously treated dimples. This is consistent with data from the open-label extension study, which show that durability for ≥1-point response on the PR-PCSS extends to 360 days for a majority (71.2%) of patients.^13,14^ Given the mechanism of the treatment, this degree of durability is not unexpected. However, patients do continue to age, and factors that confound cellulite may worsen. Furthermore, for many patients, the number of dimples to be treated may outnumber that which can be treated across 3 sessions, necessitating additional rounds of treatment. It remains to be seen whether additional rounds of treatment enhance final results or if the addition of alternate modalities proves more advantageous. As with many enzyme-based therapies, patients treated with CCH-aaes do develop antibodies to the protein.^7,15^ In the case of CCH-aaes, all patients treated were likely to develop some type of antibody response,^7^ but 66.7% of patients treated developed neutralizing antibodies to AUX-I and 78.3% developed neutralizing antibodies to AUX-II.^13^ In clinical studies, this does not appear to result in hypersensitivity or any heightened risk of AEs; however, the impact of this response on the efficacy of repeated treatments is unknown. In addition, CCH-aaes is a focal treatment for cellulite, and the safety profile for treatments beyond four sessions has not been fully established. The effect of multiple treatments to a large surface area of the buttocks, including on global shape is also unknown.

### Safety and Bruising Mitigation

The authors note that bruising, nodularity, itching, and discomfort are the most consistently reported AEs by patients, but that they generally resolve without intervention. In clinical studies of CCH-aaes, a range of AEs were monitored. However, the most notable side effect of treatment is post-injection bruising. For a small subset of patients who bruise, hemosiderin staining or prolonged hyperpigmentation can occur. The mechanism of bruising with CCH-aaes is not entirely understood, but recent anatomical studies of cellulite have shown that septae that attach to the dermis may be thicker with origins in the deep fascia or thinner (and more numerous) with origins in the superficial fascia, and that these thicker septae are associated with neurovascular bundles.^8^ It stands to reason that disruption of these thicker septae could lead to bruising. Patients should be provided with before and after photographs that clearly show not just the treatment effect but also the severity and timeline for post-injection bruising.

**Figure 6.**
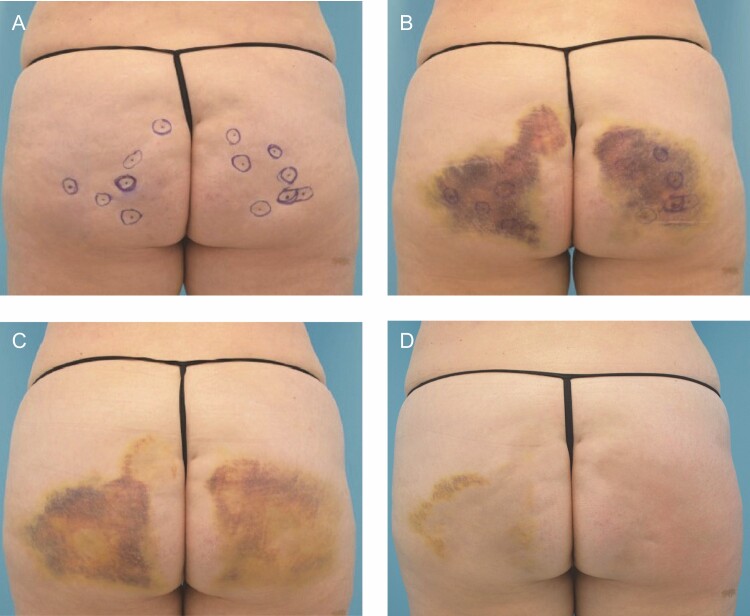
A 51-year-old female at baseline (A), and 4 days (B), 8 days (C), and 25 days (D) following treatments with collagenase *Clostridium histolyticum* (CCH-aaes) in the buttocks.

While bruising can vary widely in severity, the average trajectory of bruising is shown in Figure 6. Patients need to clearly understand the degree of bruising and the existence of treatment options to help mitigate its appearance. In addition, the risk of hemosiderin staining must be adequately considered. For patients with lighter skin, in particular, hemosiderin staining can be apparent for extended periods (from 6 months to beyond 1 year) and is less responsive to intense pulsed light (IPL) therapy or other measures that are generally successfully used to treat bruising. The risk of hemosiderin staining is one reason why proactive mitigation of bruising is so important.

During the discussion, the authors reviewed their personal experiences with various approaches for bruising mitigation. These measures are based on clinical experience alone and at the time of this writing have not been formally evaluated in clinical studies. For example, patients may be treated using the Radial Soundwave Technology (RSWT; Z-wave [Zimmier Medizin Systems, Irvine CA, USA]) 4 days after CCH-aaes injection to mitigate bruising and swelling and as a helpful adjunct for tightening skin and addressing any residual laxity. Residual bruising may also be treated with pulse dye laser treatment (PDL). Though these measures may be packaged as part of the CCH-aaes treatment, in some cases, education and reassurance for the patient that bruising is normal and will subside with time are sufficient. Formal controlled studies are needed to quantify any effect of these measures as well as whether their early use is able to reduce the risk of hemosiderin staining.

While there are no studies to support the use of ice and/or compression, these may be recommended to reduce bruising and any swelling. Patients should also be advised to avoid exercise for 24-48 hours, as there is some anecdotal evidence that increased physical activity or treatment of the same area with high-intensity focused electromagnetic energy (HIFEM) on the day of treatment worsens bruising.

**Figure 7.**
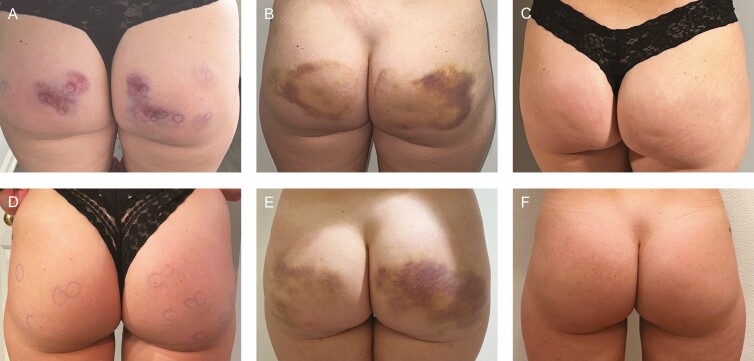
A 32-year-old twin female treated for cellulite. Oral tranexamic acid was started 48 hours after injection for twin A (A-C) and 12 hours before injection for twin B (D-F). Photographs were taken at 3-4 hours postprocedure (A, D), 6 days post injection (B, E), and 14 days post injection (C, F).

For the authors, the overall lack of preventative options prompted the consideration of tranexamic acid (TXA) as a pretreatment adjunct. Preliminary experience with this agent is described below. TXA is an antifibrinolytic agent with anti-angiogenic and antimelanogenetic properties and an established history in dermatology for the treatment of melasma, among other conditions.^16^ While TXA can be injected or applied topically, oral treatment for melasma generally requires 250 mg twice daily for 4 to 6 months. In orthopedic and trauma surgery, TXA is used to reduce perioperative bleeding, in particular for patients with bleeding disorders or taking antithrombotic medications. For plastic surgery operative procedures, it has become increasingly common to administer 1 g IV TXA at the beginning of the case to minimize postoperative ecchymosis, and some surgeons now add TXA to their liposuction tumescent solution for the same reason. Due to some anecdotal success with topical site pretreatment with TXA by one of the authors, there is research ongoing to explore the potential efficacy of a range of concentrations for reduction of bruising following treatment of cellulite with CCH-aaes. Additionally, early evidence suggests that oral TXA (1300 mg twice daily × 8 days) starting 12 hours before injection has been beneficial. In the case study shown in Figure 7, 32-year-old identical twins were treated for cellulite with CCH-aaes. Twin A was given oral TXA beginning 48 hours after injection and continued for 7 days, while Twin B was given TXA 12 hours before injection and continued for 7 days after injection. While the evidence of bruising for both twins was similar 6 days following treatment, early bruising in twin A was more severe, and the severity of bruising between injection and day 6 was higher for twin A (data not shown). While more extensive studies are certainly needed to thoroughly examine bruising risk, timeline, and severity, this case provides anecdotal evidence that TXA may reduce bruising severity. Absolute contraindications to the use of TXA include, but are not limited to, history of pulmonary embolism, deep vein thrombosis, and color blindness. Colorblindness is important as it is an indicator of toxicity. Relative contraindications include oral contraceptive use and breastfeeding.^16^

### Combination Treatments and Future Applications

As previously stated, the multifactorial contributions of cellulite along with laxity, skin quality, and localized adiposity suggest that combination therapies may yield the most optimal aesthetic results. There is no consensus on the optimal timing of skin tightening relative to CCH-aaes injections, and further research is warranted. Improving laxity before injection may permit a more precise location of actual dimples, which can improve the efficacy of injection. In contrast, if the patient desires volume augmentation, it may be more prudent to address the cellulite initially and then proceed with volumization in the form of injectable fillers or fat transfer. Skin tightening modalities can include hyperdilute biostimulatory fillers, radiofrequency, or ultrasound.

While cellulite can occur anywhere there is subcutaneous adipose tissue, it is most common in the thighs and buttocks.^4^ While none of the panelists have routinely used CCH-aaes in areas outside of the buttocks or thighs, cellulite can occur in the abdomen as well as the anterior and inner thighs. In the small number of patients that some of the authors have treated in the anterior thighs, efficacy has been good and there are no signals of heightened bruising or other safety concerns. Additional research will be needed to inform optimal injection technique and the nature of response to CCH-aaes.

Importantly, this manuscript is a reflection of the clinical experience of the authors. There is a need for evidence to further support our understanding of bruising mitigation. One shortcoming of this study is that CCH-aaes is so recently introduced to the market, that it is unclear which, if any of the presented strategies will be best for mitigation. Furthermore, additional real-world studies on outcomes for the thigh are needed, as are studies evaluating combination treatment with multiple modalities aimed at managing compounding issues such as laxity or adiposity.

## CONCLUSIONS

The introduction of CCH-aaes has been a welcome addition to the armamentarium for the treatment of cellulite. With knowledge of proper patient evaluation and injection technique, thorough patient education, diligent photography, and developing research on bruising mitigation, CCH-aaes is a minimally invasive treatment targeting septae, which shows promise as an effective and safe modality for the management of cellulite.
